# Cannabinoid Receptor 2 (CB2) Plays a Role in the Generation of Germinal Center and Memory B Cells, but Not in the Production of Antigen-Specific IgG and IgM, in Response to T-dependent Antigens

**DOI:** 10.1371/journal.pone.0067587

**Published:** 2013-06-27

**Authors:** Sreemanti Basu, Avijit Ray, Bonnie N. Dittel

**Affiliations:** 1 Blood Research Institute, BloodCenter of Wisconsin, Milwaukee, Wisconsin, United States of America; 2 Department of Microbiology and Molecular Genetics, Medical College of Wisconsin, Milwaukee, Wisconsin, United States of America; Federal University of São Paulo, Brazil

## Abstract

The cannabinoid receptor 2 (CB2) has been reported to modulate B cell functions including migration, proliferation and isotype class switching. Since these processes are required for the generation of the germinal center (GC) and antigen-specific plasma and memory cells following immunization with a T-dependent antigen, CB2 has the capacity to alter the quality and magnitude of T-dependent immune responses. To address this question, we immunized WT and CB2^−/−^ mice with the T-dependent antigen 4-hydroxy-3-nitrophenylacetyl (NP)-chicken-gamma-globulin (CGG) and measured GC B cell formation and the generation of antigen-specific B cells and serum immunoglobulin (Ig). While there was a significant reduction in the number of splenic GC B cells in CB2^−/−^ mice early in the response there was no detectable difference in the number of NP-specific IgM and IgG_1_ plasma cells. There was also no difference in NP-specific IgM and class switched IgG_1_ in the serum. In addition, we found no defect in the homing of plasma cells to the bone marrow (BM) and affinity maturation, although memory B cell cells in the spleen were reduced in CB2^−/−^ mice. CB2-deficient mice also generated similar levels of antigen-specific IgM and IgG in the serum as WT following immunization with sheep red blood cells (sRBC). This study demonstrates that although CB2 plays a role in promoting GC and memory B cell formation/maintenance in the spleen, it is dispensable on all immune cell types required for the generation of antigen-specific IgM and IgG in T-dependent immune responses.

## Introduction

The endocannabinoid system is gaining increasing recognition as an important endogenous system with an ability to fine-tune the magnitude of immune responses. One of the best-studied components of the endocannabinoid system, the cannabinoid receptor 2 (CB2) is a G_i_-protein-coupled-receptor that is abundantly expressed by immune cells, binds to the endocannabinoid 2-arachidonylglycerol (2-AG), and is thought to account for the immunomodulatory functions of this system [1]–[3]. A number of in vitro and in vivo studies demonstrated that CB2 is capable of suppressing immune responses especially involving T cells and macrophages/microglial cells suggesting that CB2 would make a good therapeutic target for the treatment of immune disorders [4]. However, experimental evidence indicates that CB2 may not be an inhibitory receptor for B cells, which express the highest level of CB2 among all immune cells [2]. In vitro studies suggest that CB2 enhances B cell proliferation and that 2-AG promotes B cell chemotaxis in a CB2-dependent manner [5], [6]. Mice deficient in CB2 have a significant reduction in marginal zone B cells that was attributed to a reduction in the homing and retention of these B cells within the marginal zone of the spleen [7]–[9]. In addition, CB2-deficient mice were shown to have impaired T-independent humoral immune responses [8], [9]. However, whether CB2 also regulates T-dependent humoral responses is not well understood.

T-dependent antibody responses are crucial for clearing complex protein antigens and generating memory against the same antigen. These responses are initiated when recirculating naïve B cells encounter specific protein antigens and become activated. Once activated, B cells interact with activated T helper cells, specific for the same antigen, which provide essential costimulatory signals for further B cell differentiation [10], [11]. Subsequently, some B cells move to the extrafollicular area, proliferate, and differentiate into short-lived plasma cells, while some B cells migrate to the follicular dendritic cell-rich area in the follicles, undergo massive proliferation, and form germinal centers (GC), where long-lived plasma and memory cells are formed [12], [13]. Early in a T-dependent response plasma cells primarily generate antigen-specific IgM. However, as the response progresses B cells undergo Ig class switching and affinity maturation and become long-lived plasma and memory B cells that home to the BM [14]–[18]. The long-lived plasma cells secrete high-affinity antigen-specific antibodies for weeks or months, and the memory cells invoke a rapid and vigorous antibody response upon a second exposure to the same antigen conferring long-term protective immunity to the host [19].

In this study, using CB2^−/−^ mice, we demonstrated that upon immunization with the T-dependent antigen 4-hydroxy-3-nitrophenylacetyl (NP)-chicken-gamma-globulin (CGG), the percentage of GC B cells was reduced in the spleen, but not lymph node. In addition, GC B cell and antigen-specific IgM and IgG_1_-secreting plasma cell formation remained unaltered in the absence of CB2. CB2-deficient mice also elicited a robust NP-specific IgM and IgG_1_ response in the serum. Furthermore, CB2-deficiency did not alter affinity maturation, and although antigen-specific memory B cells were reduced, the magnitude of the secondary antibody response was unaffected in CB2^−/−^ mice. Wild type (WT) and CB2^−/−^ mice also exhibited a comparable antigen-specific IgM and IgG response in the serum when immunized with sRBC. Together our data suggest that CB2, although known to be important for robust T-independent antibody responses, does not modulate antigen-specific T-dependent IgM and IgG responses in vivo.

## Materials and Methods

### Ethics Statement

All animal protocols used were approved by the Medical College of Wisconsin’s Institutional Animal Care and Use Committee. We monitored immunized animals for adverse health issues and used appropriate methods of euthanasia.

### Mice and Reagents

The generation of WT (CB2^+/+^) and CB2^−/−^ mice on the C57BL/6 background has been previously described [9]. Mice were maintained in the Translational Biomedical Research Center of the Medical College of Wisconsin. Anti-mouse B220-PE-Texas Red (RA3–6B2), Fas-PE (Jo2), GL-7-FITC (GL7), and IgG_1_-FITC (A85–1) were purchased from BD Biosciences (San Jose, CA). Anti-CD38-AlexaFluor 647 (90) and anti-IgD-Pacific blue (11–26c.2a) were obtained from BioLegend (San Diego, CA). NP-CGG, NP-BSA, and NP-PE were purchased from Biosearch Technologies (Novato, CA) and sRBC were purchased from Colorado Serum Company (Denver, CO).

### Immunization and Serum Collection

For primary humoral responses, 6–12 week, age- and sex-matched WT and CB2^−/−^ mice were immunized with 30 µg alum-precipitated NP-CGG with a NP to CGG ratio of 27∶1 (i.p. or s.c. on the shoulders) or with 1×10^8^ sRBC (i.p.). For secondary humoral responses, mice were rechallenged with 30 µg of NP_27_-CGG resuspended in PBS on day 42 after the primary immunization with the same antigen. Serum were collected on the indicated time points to assess antigen-specific IgM, IgG, and IgG_1_ titers.

### Flow Cytometry

Single cell suspensions were prepared from spleens, brachial lymph nodes (LN), and bone marrow (BM) collected from both femurs. 1×10^6^ splenocytes and brachial LN cells were stained with anti-B220, anti-Fas and anti-GL7 to analyze B220^+^Fas^+^GL7^+^ GC B cells by flow cytometry. For memory B cell analysis, 10–20×10^6^ splenocytes and BM cells were labeled with NP_8_-PE and appropriate fluorochrome-conjugated anti-mouse B220, IgD, IgG_1_ and CD38. B220^+^NP_8_^+^IgD^−^IgG_1_^+^CD38^+^ cells represented NP-specific high-affinity class-switched memory B cells. Samples were acquired with a LSR II flow cytometer (Becton Dickinson, San Jose, CA) and data were analyzed using FlowJo software (Tree Star, Ashland, OR).

### ELISPOT

For the detection of antigen-specific antibody secreting plasma cells 96 well MultiScreen_HTS_ Filter Plates (Millipore, Bedford, MA) were coated with 5 µg NP_25_-BSA overnight at 4°C. To assess high affinity antibody secreting cells, ELIPOT plates were coated overnight with 5 µg NP_4_-BSA at 4°C. Nonspecific binding was blocked with 10% fetal calf serum for 2 h at room temperature. Subsequently, splenocytes, BM or LN cells (RBC depleted) were added and incubated at 37°C for 3–4 h. NP-specific IgM and IgG_1_ spots were detected using HRP-conjugated anti-IgM or anti-IgG_1_ and the 3-amino-9- ethyl-carbazole substrate (Sigma-Aldrich, St. Louis, MO).

### ELISA

To determine antigen-specific serum IgM and IgG_1_ titers, 96 well flat-bottom Nunc MaxiSorp® Microwell plates were coated overnight with 5 µg NP_25_-BSA or with 5 µg NP_4_-BSA for the detection of high affinity antibodies diluted in carbonate buffer. The plates were blocked with 1% BSA for 2 h at room temperature prior to the addition of serially diluted serum samples. Subsequently, HRP-conjugated anti-IgM and anti-IgG_1_ were added to the wells and NP-specific IgM and IgG_1_ levels were detected using 2,2′-Azinobis (3-ethylbenzothiazoline-6-sulfonic acid)-diammonium salt (ABTS) substrate (Southern Biotechnology, Birmingham, AL). The sRBC-specific IgM and IgG ELISA were performed as described [20]. Briefly, 96 well flat-bottom MaxiSorp Microwell plates were coated overnight with 5×10^6^ sRBC diluted in PBS at 4°C and then following an incubation with 0.3% glutaraldehyde for 30 min at 25°C, the plates were washed and serially diluted serum samples were added. Antigen-specific IgM and IgG were detected using HRP-conjugated anti-IgM and anti-IgG and ABTS substrate. Absorbance was measured at 405 nm.

### Statistical Analysis

A two-tailed nonparametric Mann-Whitney Test was used to determine statistical significance. A p-value <0.05 was considered statistically significant.

## Results and Discussion

### I.p. Immunization of CB2^−/−^ Mice with NP-CGG Results in a Reduction in the Percentage of GC B Cells in the Spleen with no Alteration in Antigen-specific Ig Production

**Figure 1:**
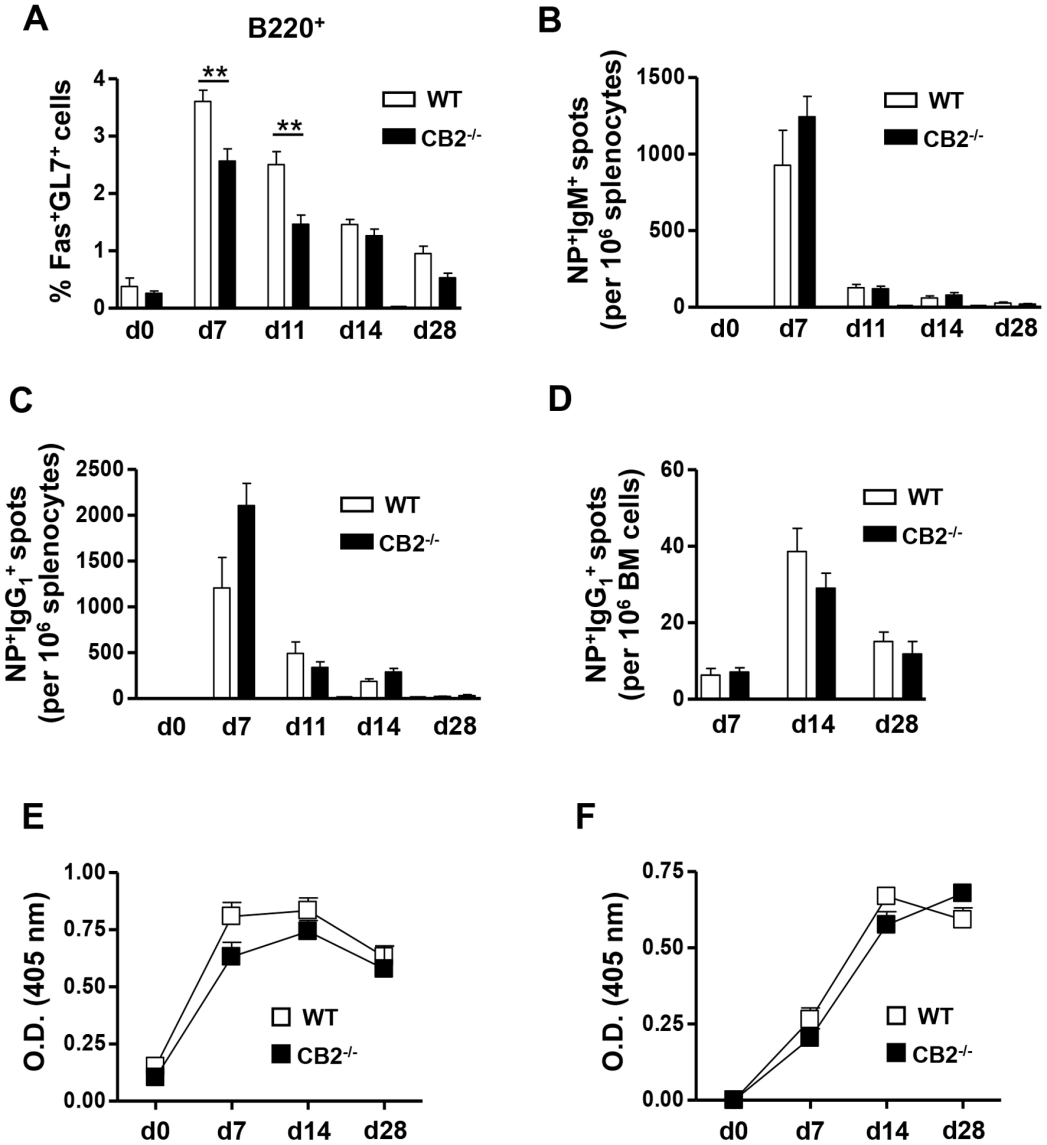
GC B cells are reduced, but antigen-specific plasma B cell formation and antigen-specific serum IgM and IgG_1_ levels are unaltered in CB2^−/−^ mice upon i.p. immunization with a T-dependent antigen. WT (open bars) and CB2^−/−^ (closed bars) mice were i.p. immunized with 30 µg alum-precipitated NP-CGG. (A–D) Spleens and BM were collected before (day 0) or 7, 11, 14, and 28 days after immunization for the analysis of GC B cells in the spleen by flow cytometry (A) and NP-specific IgM and IgG_1_-secreting plasma cells in the spleen (B, C, respectively) and BM (D) by ELISPOT. (A) The percentage of Fas^+^GL7^+^ GC B cells within the B220^+^ population is shown. (B–D) Frequencies of NP-specific IgM^+^ (B) and IgG_1_^+^ spots (C) per 10^6^ splenocytes and NP-specific IgG_1_^+^ spots per 10^6^ BM cells (D) on the indicated days are shown. (E, F) Serum was collected from NP-CGG-immunized WT and CB2^−/−^ mice on days 0, 7, 14 and 28 and NP-specific IgM and IgG_1_ titers were measured from two-fold serially diluted serum (1/400 to 1/3200 for IgM and 1/20000 to 1/160000 for IgG_1_) by ELISA. The O.D. value for NP-specific IgM at the 1/1600 dilution (E) and NP-specific IgG_1_ at the 1/40000 dilution (F) are shown. Data shown are the mean ± SEM from two independent experiments each with 3–4 mice per group. **p<0.01.

We recently reported that CB2^−/−^ mice have a significant reduction in T-independent immune responses [9] and in an extension of those studies, here we determined whether CB2 regulates T-dependent immune responses. Since the generation of a stable GC where long-lived high-affinity plasma cells are formed is a hallmark of T-dependent humoral responses, we first asked whether the GC is formed normally in CB2^−/−^ mice. In WT mice that were i.p. immunized with the T-dependent antigen NP-CGG, the number of GC B cells (Fas^+^GL7^+^) in the spleen peaked on day seven and then contracted gradually on days 11, 14 and 28 (Fig. 1A). Interestingly, we found a significant reduction in the percentage of GC B cells in CB2^−/−^ mice on days seven and 11 (Fig. 1A). However, there was no difference at later time points (days 14 and 28) (Fig. 1A). Next, we assessed whether the reduction in the percentage of GC B cells in CB2^−/−^ mice correlated with reduced antigen-specific plasma cell formation. To our surprise, we observed no significant difference in the frequencies of NP-specific IgM (Fig. 1B) or IgG_1_-secreting plasma cells (Fig. 1C) in the spleen between WT and CB2^−/−^ mice after NP-CGG immunization (i.p.). The antigen-specific IgG_1_-secreting plasma cells homed to the BM comparably in CB2^−/−^ and WT mice (Fig. 1D). In addition, CB2^−/−^ mice exhibited similar NP-specific serum IgM (Fig. 1E) and IgG_1_ (Fig. 1F) levels post-primary immunization as compared to WT mice.

Interestingly, we found no difference in the generation of NP-specific isotype class switched IgG_1_ plasma cells indicating that CB2 does not regulate immunoglobulin class switching to IgG. However, we did not study other Ig isotypes. This is of interest because it was reported that CB2^−/−^ mice generated higher levels of total IgE in the serum compared to WT mice after immunization with the T-dependent antigens ovalbumin (in alum) or KLH (in RIBI), suggesting a role for CB2 in the down regulation of serum IgE [21]. However, whether CB2 regulates the generation of antigen-specific serum IgE was not investigated. Agudelo and colleagues also reported that the synthetic cannabinoid CP55940 increased IgM to IgE class switching in vitro, which was reversed by the addition of the CB2-selective antagonist/inverse agonist SR144528, suggesting a role for CB2 [22]. However, in a similar study, the CB2 selective agonist Gp1a decreased IgE production, which was also reversed by SR144528 [21]. These contradictory results highlight the complexity of the cannabinoid system, where cannabinoids or synthetic agonists can bind and function through multiple receptors [4]. Therefore, as in our study, the use of CB2^−/−^ mice is an important tool for gaining a better understanding of the role for CB2 in humoral immune responses.

### CB2^−/−^ Mice Immunized with NP-CGG via a s.c. Route Exhibit No Alteration in the Frequencies of GC B Cells and Antigen-specific Antibody-secreting Plasma Cells in the Draining LN or in Serum Antigen-specific Ig Levels

**Figure 2:**
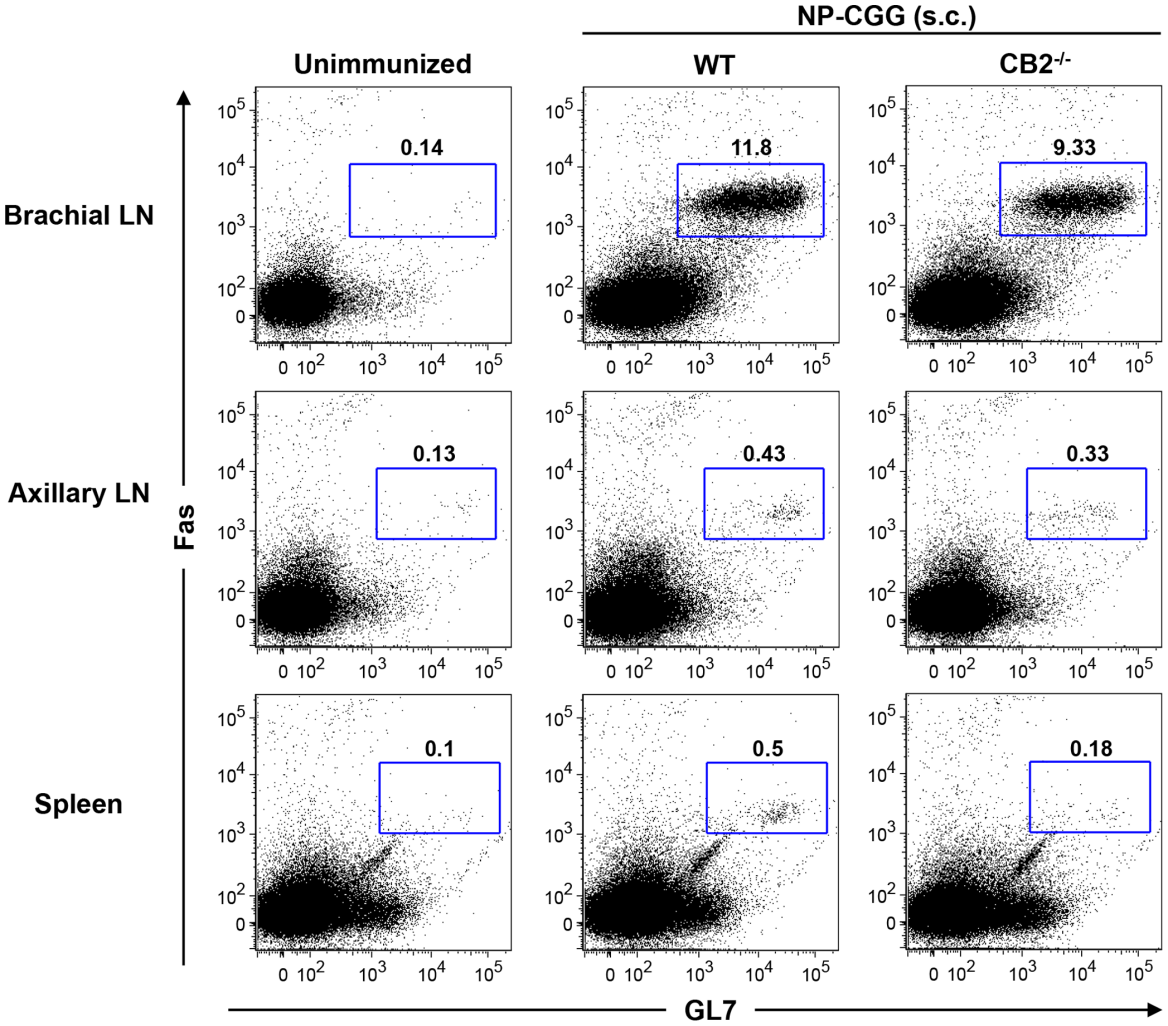
Upon s.c. immunization with NP-CGG GC formation occurs primarily in the draining LN. WT and CB2^−/−^ mice were s.c. immunized with 30 µg alum-precipitated NP-CGG s.c. on the shoulders. On day seven, the draining brachial and non-draining axillary LN and the spleen were collected and GC B cells were analyzed by flow cytometry. Representative dot plots show relative frequencies of Fas^+^GL7^+^ GC B cells within the B220^+^ population in the brachial LN (top row), axillary LN (middle row), and the spleen (bottom row) from unimmunized controls (left panels), immunized WT (middle panels) and CB2^−/−^ (right panels) mice. Numbers on the plots represent the percentage of cells in the corresponding gate. Data shown are one representative experiment of three.

**Figure 3:**
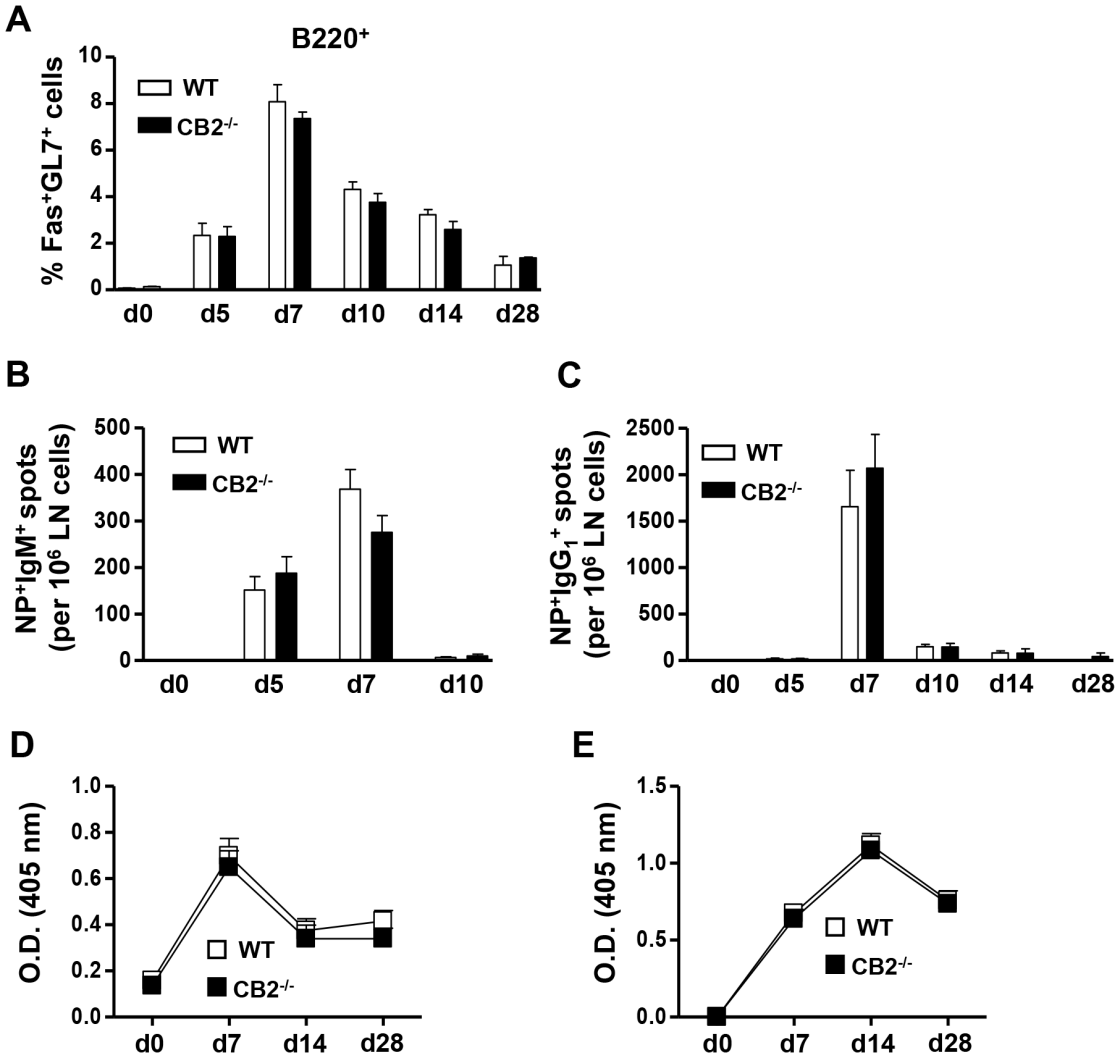
Upon s.c. NP-CGG immunization CB2^−/−^ mice elicit normal GC B cell and antigen-specific IgM and IgG_1_ responses. WT (open bars) and CB2^−/−^ (closed bars) mice were s.c. immunized with 30 µg alum-precipitated NP-CGG. (A–C) On day 0 or days 5, 7, 10, 14, and 28 post-immunization, the draining LN were collected and GC B cells were analyzed by flow cytometry (A) and NP-specific IgM and IgG_1_ secreting plasma cells were determined by ELISPOT (B, C, respectively). (A) The percentage of Fas^+^GL7^+^ GC B cells within the B220^+^ population is shown. (B, C) Frequencies of NP-specific IgM^+^ (B) and IgG_1_^+^ spots (C) per 10^6^ LN cells are shown. (D, E) NP-specific IgM and IgG_1_ titers were measured from two-fold serially diluted serum (1/200 to 1/1600 for IgM and 1/10000 to 1/80000 for IgG_1_) from NP-CGG-immunized WT and CB2^−/−^ mice on days 0, 7, 14, and 28 by ELISA. The O.D. values for NP-specific IgM at the 1/800 dilution (E) and NP-specific IgG_1_ at the 1/40000 dilution (F) are shown. Data shown are the mean ± SEM from two independent experiments each with 3 mice per group (n = 6).

In i.p. immunizations the primary humoral response largely occurs in the spleen, which has a different architecture and cellular content as compared to the LN. The spleen contains a marginal zone area that lines the white and the red pulp in which resides a unique B cell subset called marginal zone B cells. These B cells express a high level of CB2 and are believed to participate in T-dependent humoral responses [8], [9], [23]–[25]. Since, CB2^−/−^ mice have reduced numbers of marginal zone B cells, it is possible that CB2 regulates the magnitude of humoral responses differently in the spleen and LN. To test this possibility, we s.c. immunized WT and CB2^−/−^ mice with NP-GCC and determined the percentage of GC B cells in the draining LN (brachial), non-draining LN (axillary) and the spleen seven days later. As shown in Fig. 2, the generation of GC B cells was largely restricted to the draining LN in both WT and CB2^−/−^ mice. Unlike the spleen with i.p. immunization (Fig. 1A), no difference in the percentage of GC B cells in the draining lymph node of WT and CB2^−/−^ mice was detected over 28 days (Fig. 3A). We also observed no difference in the frequency of NP-specific IgM (Fig. 3B) and IgG_1_-secreting plasma cells (Fig. 3C). Similarly, antigen-specific IgM (Fig. 3D) and IgG_1_ (Fig. 3E) levels in the serum were unaltered in CB2^−/−^ mice.

Collectively, these data suggest that CB2 is neither essential for nor does it regulate the magnitude of the primary T-dependent IgM and IgG_1_ responses upon NP-CGG immunization. This finding is in agreement with the observation by Muppidi, et al., that in mixed chimera mice generated with a 1∶1 ratio of WT and CB2^−/−^ BM similar levels of antigen-specific IgG_1_ from each donor mouse was present in the serum 12 days after immunization with a T-dependent antigen [8]. Thus CB2 regulates the magnitude of T-independent but not -dependent humoral responses. The explanation for attenuated T-independent immune responses in CB2^−/−^ mice is likely due to reduced numbers of marginal zone B cells, which play a critical role in eliciting antibody responses against T-independent antigens [7]–[9]. Since follicular B cell numbers, the central players of a T-dependent humoral response [7], [9], are unaffected in the absence of CB2 the magnitude of the T-dependent antibody responses are also unaltered in CB2-deficient mice.

In this study, a contrasting observation was the reduction in the percentage of GC B cells in the spleen (during i.p. immunization), but not in the brachial LN (during s.c. immunization). This may also be explained by reduced numbers of marginal zone B cells in CB2^−/−^ mice since the marginal zone is only present in the spleen. During i.v. or i.p. immunization, marginal zone B cells can encounter antigens, and participate in T-dependent responses. These cells are capable of forming GC [23]. In addition, marginal zone B cells are potent activators of CD4 T cells, which play an essential role in GC formation [24]. Therefore, it is possible that the diminished percentage of GC B cells on days seven and 11 after i.p. NP-CGG immunization in CB2^−/−^ mice reflects lower numbers of marginal zone B cells able to contribute to the GC B cell pool. However, it is also possible that this reduction is due to defects in other cellular compartments in the spleen of CB2^−/−^ mice.

### CB2-deficiency does not Alter Affinity Maturation

**Figure 4:**
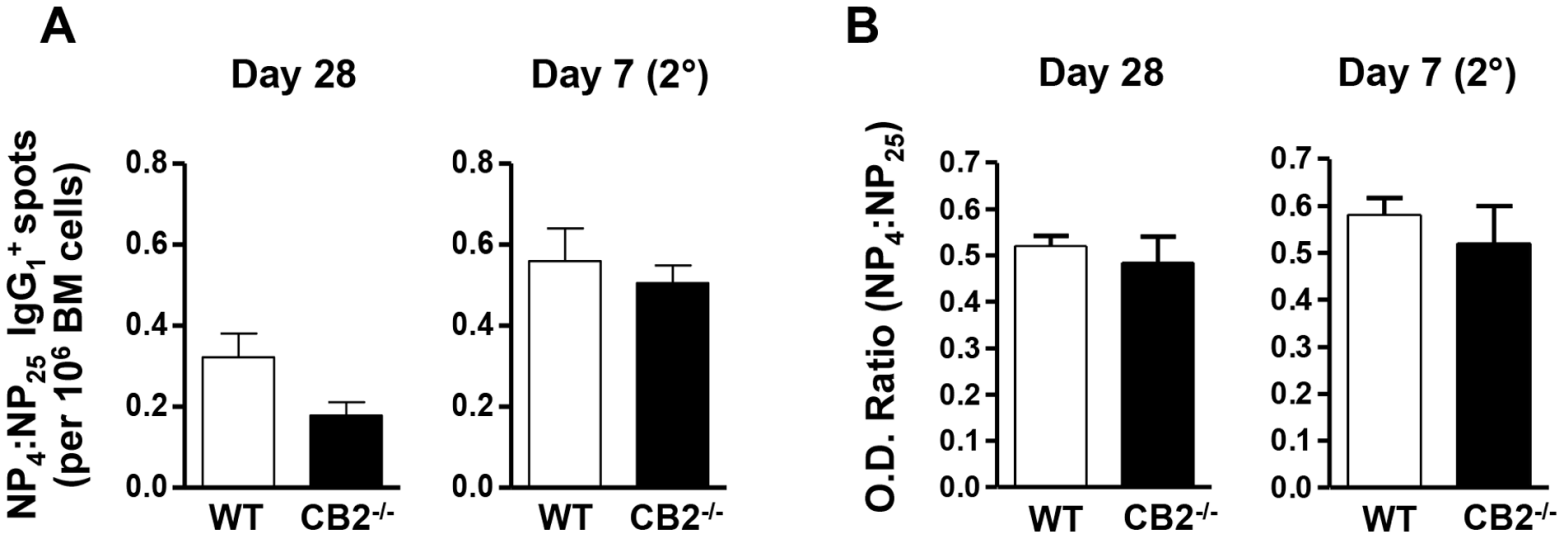
CB2-deficiency does not affect affinity maturation. WT (open bars) and CB2^−/−^ (closed bars) mice were immunized with 30 µg NP-CGG in alum (i.p.) and rechallenged with 30 µg of NP-CGG in PBS (i.p.) on day 42 post-primary immunization. BM and serum were collected on day 28 after the primary and day seven after the secondary immunization for the analysis of affinity maturation. (A) Frequencies of high affinity (NP_4_-specific) and total (NP_25_-specific) antigen-specific IgG_1_-secreting plasma cells per 10^6^ BM cells were determined by ELISPOT. Affinity maturation calculated as a ratio of high affinity to total antigen-specific IgG_1_-secreting cells (NP_4_:NP_25_) is shown. (B) The titers of high affinity (NP_4_-specific) and total (NP_25_-specific) antigen-specific IgG_1_ were determined from two-fold serially diluted serum (1/20000 to 1/160000) by ELISA and the O.D. values at the 1/40000 dilution were used to calculate affinity maturation. Affinity maturation calculated as an O.D. ratio of high affinity to total antigen-specific IgG_1_ (NP_4_:NP_25_) is shown. Data shown are the mean ± SEM from two independent experiments each with 2–4 mice per group (n = 6–7).

We next investigated whether CB2 controls the quality of a T-dependent antibody response by regulating affinity maturation. We immunized WT and CB2^−/−^ mice i.p. with NP-CGG on day 0 and rechallenged i.p. on day 42, and analyzed affinity maturation on day 28 after the primary and on day seven after the secondary immunization. At both time points, we observed no statistically significant difference in affinity maturation between WT and CB2^−/−^ mice (Fig. 4A, B). In the s.c. immunization affinity maturation was also comparable between WT and CB2^−/−^ mice (data not shown).

From these data we concluded that CB2 plays little or no role in GC reactions that involve the orchestration of a variety of cell types. This finding is particularly important in the context of B cell migration since positioning within the GC is critical to facilitate T:B cell interactions required for both isotype class switching and affinity maturation. It is known that the endocannabinoid 2-AG promotes the migration of B cells including the follicular subset [5], [6], [9]. Thus while CB2 is critical for the homing of marginal zone B cells [8], [9], it doesn’t seem to play a similar role in the positioning of follicular B cells in the GC.

### CB2 Alters Memory B Cell Numbers in the Spleen, but not BM, following a Secondary Challenge in the Absence of Changes in Plasma Cell Numbers and Antigen-specific Ig in the Serum

**Figure 5:**
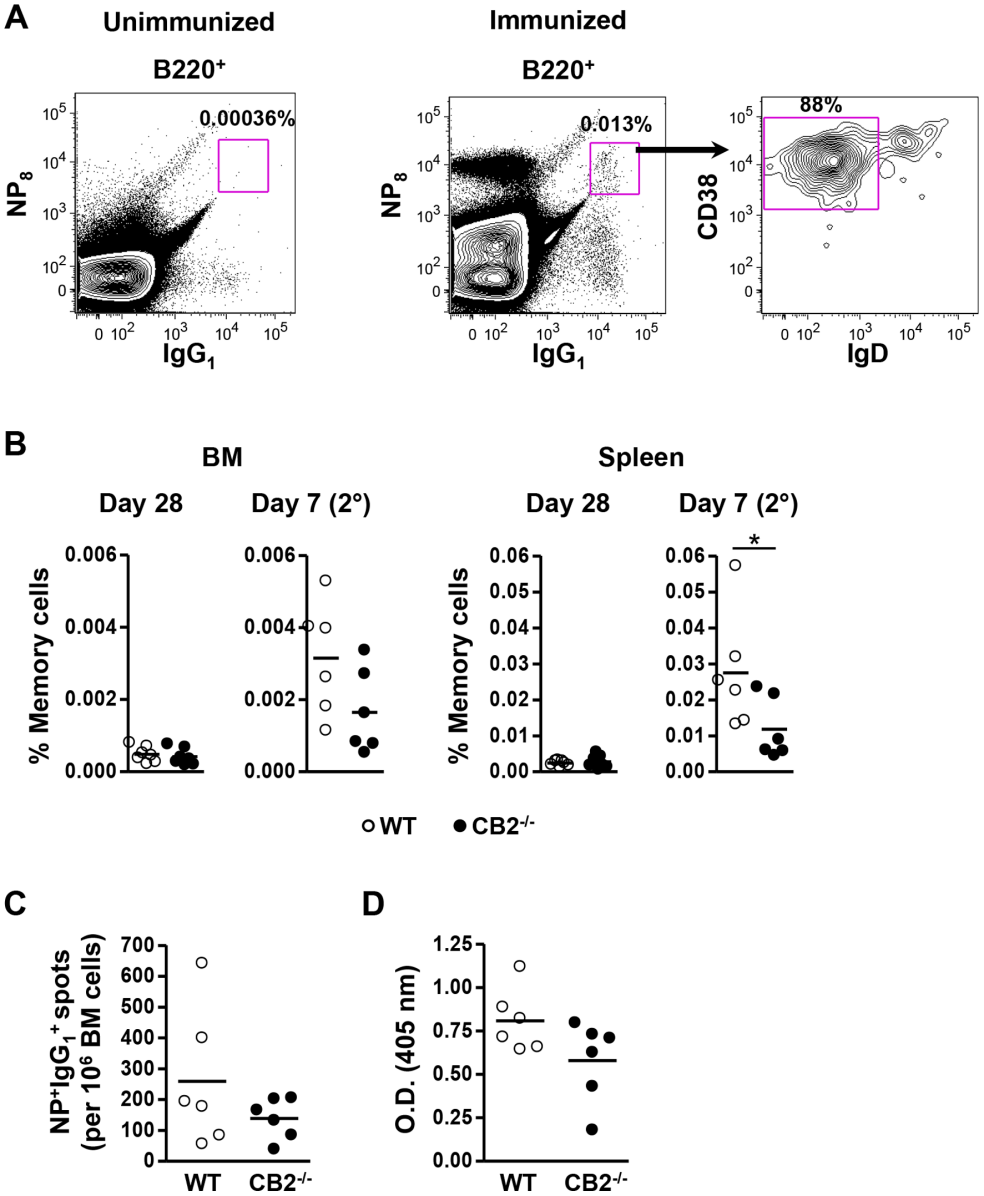
Memory B cell formation is reduced while the secondary humoral response after NP-CGG immunization is unaffected in CB2^−/−^ mice. WT and CB2^−/−^ mice were immunized (i.p.) with 30 µg alum-precipitated NP-CGG and rechallenged with 30 µg of NP-CGG in PBS (i.p.) on day 42 post-primary immunization. (A,B) BM and spleen were collected on day 28 after the primary and day seven after the secondary immunization for the analysis of memory B cells by flow cytometry. (A) Representative flow cytometric plots show the gating strategy for memory B cells. B220^+^ cells were analyzed for NP_8_ and IgG_1_ in unimmunized (left panel) and in immunized (middle panel) mice and NP_8_^+^IgG_1_^+^ cells were further analyzed for the expression of IgD and CD38 (right panel). The B220^+^NP_8_^+^IgG_1_^+^IgD^−^CD38^+^ population represented antigen-specific high-affinity class-switched memory B cells. (B) The cumulative percentage of memory B cells within the lymphocyte population of the BM and spleen is shown. (C, D) On day seven after the secondary immunization the frequency of NP-specific IgG_1_-secreting plasma cells in the BM was determined by ELISPOT (C) and the NP-specific serum IgG_1_ titer was measured from two-fold serially diluted serum (1/40000–1/320000; O.D. at 1/160000 the dilution is shown) by ELISA (D). (B–D) Data are represented as the mean ± SEM from two independent experiments each with 2–4 mice per group (n = 6–7). *p<0.05.

Because memory B cells generated during T-dependent responses rapidly expand and differentiate into plasma cells eliciting a rapid and robust secondary response upon a secondary challenge with the same antigen, we evaluated whether CB2-deficiency affects memory B cell formation. This was determined by rechallenging mice with NP-CGG 42 days after the primary immunization and determining the percentage of antigen-specific memory cells seven days later. Figure 5A demonstrates our gating strategy based on published studies [26] in which B220-gated cells were subsequently gated on IgG_1_^+^NP_8_^+^ cells. In the control unimmunized mice there were few IgG_1_ class switched B cells and among these there was only negligible NP_8_ binding as compared to immunized mice (Fig. 5A, left panel versus middle panel). The IgG_1_^+^NP_8_^+^ cells were further separated by CD38 and IgD with the memory B cells having a B220^+^NP_8_^+^IgD^−^IgG_1_^+^CD38^+^ phenotype (Fig. 5A, right panel). We found similar percentages of high-affinity class-switched memory B cells in WT and CB2^−/−^ mice in the BM and spleen 28 days after the primary immunization (Fig. 5B). The mice were then rechallenged on day 42 and seven days later there was no significant difference in the percentage of memory B cells in the WT (0.0032±0.0066) versus CB2^−/−^ (0.0017±0.0005) mice in the BM (Fig. 5B). In contrast, there was a significant reduction in the percentage of memory cells in the spleen of CB2^−/−^ (0.28±0.007) versus WT (0.12±0.004) mice (Fig. 5B). These data indicate that CB2 plays a role in the generation or maintenance of memory cells. While the mechanism of this regulation was not elucidated, we hypothesize that in the absence of CB2 memory cells are not retained within the spleen, likely due to diminished migration/retention to CB2 agonists present in the spleen. Interestingly, CB2 does not seem to similarly contribute to the memory pool in the BM suggesting that their retention within the BM is not regulated by CB2.

Next, we assessed whether the secondary humoral response is altered in the absence of CB2. On day seven after NP-CGG rechallenge (i.p.), we observed a robust expansion of NP-specific IgG_1_-secreting plasma cells in the BM (Fig. 1D versus Fig. 5C) in WT mice. However, when we compared the frequency of NP-specific IgG_1_-secreting plasma cells between WT (259±91) and CB2^−/−^ (139±27) mice in the BM there was not a significant difference (Fig. 5C). Similarly, CB2^−/−^ (0.58±0.1) mice elicited a similar level of antigen-specific IgG_1_ in the serum as the WT (0.81±0.07) on day seven after secondary immunization (Fig. 5D). Interestingly, while a CB2 deficiency resulted in a significant decrease in the number of memory B cells in the spleen (Fig. 5B) there was no parallel decrease in the number of antibody secreting cells (Fig. 5C). Thus CB2 seems to regulate memory, but not, plasma cell numbers. Given the known functions of CB2 in the immune system, the reason for this finding could be due to differential regulation of migration, survival or proliferation by CB2 in unique B cell subsets.

### CB2^−/−^ Mice Elicit a Normal Humoral Response Against sRBC

**Figure 6:**
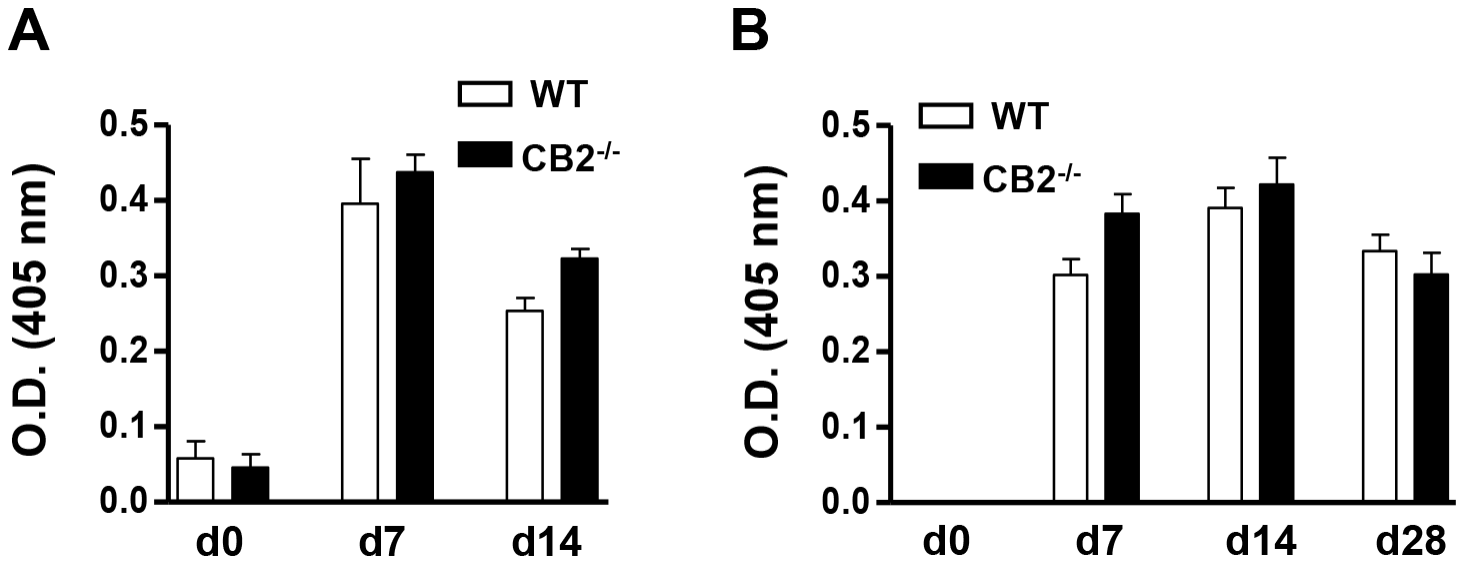
WT and CB2^−/−^ mice elicit comparable antigen-specific IgM and IgG responses in the serum after sRBC immunization (i.p.). WT and CB2^−/−^ mice were immunized (i.p.) with 1×10^8^ sRBC and serum was collected on days 0 (pre-immunization), 7, 14, and 28. Antigen-specific IgM and IgG titers were measured from three-fold serially diluted (1/50 to 1/1350 for IgM and 1/150 to 1/4050 for IgG) serum samples by ELISA. The O.D. values for sRBC-specific IgM at the 1/50 dilution (A) and sRBC-specific IgG at the 1/450 dilution (B) are shown. Data shown are the mean ± SEM from two independent experiments each with 3–4 mice per group (n = 6–8).

To further assess the role of CB2 in T-dependent humoral responses, we chose to study another classically used T-dependent antigen sRBC. Upon i.p. immunization with sRBC, CB2^−/−^ mice elicited antigen-specific IgM and IgG responses in the serum similarly to WT mice (Fig. 6A and 6B, respectively).

Using a variety of approaches and multiple T-dependent antigens, we have concluded that while CB2 does play a role in GC and memory B cell formation/maintenance in the spleen, it does not regulate the quality or the magnitude of the T-dependent humoral response in vivo. Since these studies were carried out in a global CB2-deficient mouse, this finding indicates that CB2 is dispensable on all immune cells required for the generation of effector molecules that invoke signaling events leading to isotype class switching, affinity maturation and the generation of long-lived plasma cells. To the best of our knowledge this study is the first to quantitate antigen-specific B cell numbers in CB2^−/−^ mice following immunization with a T-dependent antigen. However, similar to our findings, when CB1^−/−^CB2^−/−^ mice were immunized with sRBC and the number of sRBC-specific IgM splenic antibody forming cells was measured 4 days later there was no significant difference between WT and the double knockout mice [27]. In a second study, CB2^−/−^ mice were immunized with the T-dependent antigen ovalbumin, and when antigen-specific IgM was measured in the serum 12 days later there was no significant difference between WT and CB2^−/−^ mice [8]. These cumulative findings suggest that while collectively the cannabinoid system is considered immunosuppressive, the administration of CB2-selective agonists for the treatment of inflammatory diseases, such as autoimmunity, should not alter B cell-specific immunity to pathogens.
